# Immaturity of Ganglia in Familial Onset: Three Cases of Twins and Their Brother

**DOI:** 10.70352/scrj.cr.25-0092

**Published:** 2025-11-07

**Authors:** Shotaro Taki, Yoshizo Kimura, Hiroto Eto, Shiori Tsuruhisa, Tomohiro Kurahachi, Daisuke Masui, Motomu Yoshida, Hiroaki Tanaka, Koichi Higaki, Takahiro Asakawa, Kimio Asagiri

**Affiliations:** 1 Department of Pediatric Surgery, St. Mary’s Hospital, Kurume, Fukuoka, Japan; 2 Department of Pathology, St. Mary’s Hospital, Kurume, Fukuoka, Japan; 3 Department of Pediatric Surgery, Kurume University School of Medicine, Kurume, Fukuoka, Japan

**Keywords:** immaturity of ganglia, allied disorders of Hirschsprung’s disease, twins, case report

## Abstract

**INTRODUCTION:**

Immaturity of ganglia (IG) is a rare disease and is classified as a type of allied disorders of Hirschsprung’s disease (HSCR). Recently, familial occurrence of HSCR has often been reported. However, there have been very few reports of familial occurrence of IG. We report 3 cases of intrafamilial occurrence of IG.

**CASE PRESENTATION:**

Case 1 was an older brother. He was born vaginally at 40 weeks and 6 days of gestation, weighing 3658 g. No prenatal diagnosis was made. On the day of birth, abdominal distention appeared, and a gastric tube was inserted. On the 1st day of life, colonography showed microcolons throughout the colon. We diagnosed gastrointestinal obstruction or Hirschsprung’s disease and performed surgery on the same day. Cases 2 and 3 were monozygotic twins. They were born vaginally at 37 weeks and 0 days, weighing 2778 and 2810 g, respectively. Neither of them had a prenatal diagnosis. On the 1st day of life, abdominal distention, malfeeding, and delayed evacuation of feces were observed, and colonography was performed. Due to the presence of microcolons throughout the colon, we decided to operate on them. In all 3 cases, ileostomies were created, and the stomas were closed after 6 months. They began oral intake and infusion early on, and anal defecation was established. Also, immature ganglion cells were confirmed by HuC/D staining during the 1st operation. At the time of stoma closure, we confirmed that ganglion cells had matured.

**CONCLUSIONS:**

IG, like HSCR, may have intrafamilial onset. Therefore, early diagnosis and treatment planning are important. Also, a careful explanation to the family is essential.

## Abbreviations

ADHD
: allied disorders of Hirschsprung’s disease
HE
: hematoxylin and eosin
HSCR
: Hirschsprung’s disease
IG
: immaturity of ganglia

## INTRODUCTION

IG is a rare disease and is classified as a type of ADHD. Recently, familial occurrence of HSCR has often been reported. However, there have been very few reports of familial occurrence of IG, with only 1 previous report^1)^ and no other references. We report 3 cases of intrafamilial occurrence of IG.

### The mother’s background

There was no family history. She was G2P0 and had 1 spontaneous miscarriage, as well as a history of abortion due to a fetal umbilical cord hernia at 14 weeks of gestation.

## CASE PRESENTATION

### Case 1: Older brother

**Fig. 1:**
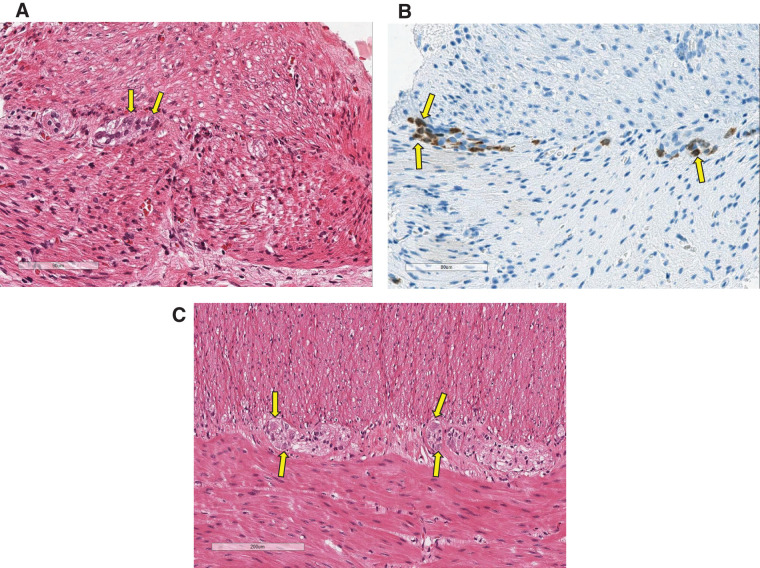
Pathological findings of the older brother. (**A**) HE staining: Ganglion cells were present, but very few and immature (arrows). (**B**) HuC/D staining: Many more ganglion cells were observed than with HE staining, but they were still immature (arrows). (**C**) HE staining: Mature and enlarged ganglion cells were observed (arrows). The nerve plexus was larger than before. HE, hematoxylin and eosin

We present a table of the characteristics of the 3 patients (**Table 1**). The older brother was born at 40 weeks and 6 days of gestation and was treated with bicuculline due to a water breakage in the 1st trimester. An emergency cesarean section was performed for cephalopelvic disproportion. He was born weighing 3658 g. On the day of birth, abdominal distention was observed. White stools were observed, raising suspicion of a gastrointestinal obstructive condition. On day 1, contrast enema revealed a 30-cm fecal impaction from 150 to 180 cm. A 25-cm intestinal segment (145–170 cm) and the appendix were resected for pathology, and a double-barrel ileostomy was created 170 cm distal to the ligament of Treitz. Pathological examination with HE staining showed a decrease in the intermuscular plexus and ganglion cells. However, HuC/D staining showed no evidence of ganglion cell reduction (**Fig. 1A** and **1B**); furthermore, these ganglion cells were immature. He was started on breastfeeding from the 4th POD, and we began injecting into the anal side of the stoma on the 22nd POD. A small amount of breast milk was used for the 1st injection, and the volume was gradually increased. Finally, the drainage fluid collected from the oral-side stoma was injected into the anorectal stoma. Two hundred eighty-five days after the ileostomy, when oral feeding and anal-side defecation had stabilized, colostomy closure was performed at a weight of 8190 g. After stoma closure, the patient was able to defecate anally and was doing well. Pathological examination with HE staining showed mature ganglion cells and led to the diagnosis of IG (**Fig. 1C**).

**Table 1:** Background of patients

	Case 1	Case 2	Case 3
	Older brother	Older twin	Younger twin
Sex	M	F	F
GA	40 w, 6 d	37 w, 0 d	37 w, 0 d
Birth weight (g)	3658	2778	2810
Age at diagnosis (days)	1	1	1
Stoma type	Double-barrel ileostomy	Double-barrel ileostomy	Double-barrel ileostomy
Nutrition of the anal side stoma	+	+	+
Age at stoma closure (days)	285	205	205
Weight at stoma closure	8190	7760	6730
Mature ganglion cells at stoma closure	+	+	+

The table shows the characteristics of the older brother, the older female twin, and the younger female twin.

d, days; F, female; GA, gestational age; M, male; w, weeks

### Case 2: Older female twin

The mother was pregnant with monozygotic twins and underwent a planned cesarean section at 37 weeks and 0 days of gestation, 2 years after the older brother’s surgery. The older female twin weighed 2778 g at birth. On the 1st day of life, abdominal distention and delayed evacuation of feces were observed, and colonography showed the same findings as her older brother. Due to the presence of microcolons throughout the colon, we suspected a condition similar to that of her older brother.

A 40-cm segment of the ileum located 10 cm proximal to the ileocecal valve was resected, and a double-barrel ileostomy was created. Pathological examination showed ganglion cells on the oral side, with no abnormalities in number and no compensatory changes in the plexus. On the anal side, areas with few ganglion cells were observed, and a part of the plexus was small. However, like her brother, HuC/D staining showed no evidence of ganglion cell reduction, which led to the diagnosis of IG. She was started on breastfeeding from the 2nd POD. The stoma opening became temporarily narrowed, so parenteral nutrition and nutritional infusion into the anal-side stoma were continued. At around 6 months of age, colostomy closure was performed at a body weight of 7760 g. Pathological examination showed mature ganglion cells, confirming the diagnosis of IG.

### Case 3: Younger female twin

She was born weighing 2810 g. She had the same findings as her sister and therefore underwent surgery. Due to intestinal dilatation and fecal impaction for 15 cm from 65 cm from the ileocecal valve, the dilated 50 cm intestinal tract was resected, and a double-barrel ileostomy was created. The pathological findings were similar to those of her sister. She also underwent colostomy closure at around 6 months of age at a body weight of 6730 g. The pathological findings at that time were similar to those of her 2 other siblings.

## DISCUSSION

IG does not have a clear worldwide definition, and Japan has intermittently proposed a definition of ADHD. A Japanese ADHD study group proposed pathological criteria based on HE staining: (a) immaturity of ganglion cells (small size of ganglion cells) and (b) a normal number and distribution of ganglion cells. A combination of (a) and (b) is necessary for the pathological diagnosis of IG.^1)^ In addition to this definition, the maturation of immature ganglion cells is considered an indicator. In this study, we focused on cell body enlargement to determine “maturity” using HuC/D staining, which has recently been reported for its usefulness.^1,2)^ In our 3 cases, we were able to confirm the maturation of immature ganglion cells at the time of colostomy closure. Immature ganglion cells are proposed to mature over time,^3)^ as evidenced by the pathological results at the time of stoma closure in these cases.

It was previously noted that HuC/D staining may be useful for confirming immature ganglion cells. In our experience, we were not able to confirm the immature ganglion cells with HE staining, but instead were able to confirm them with HuC/D staining, demonstrating the usefulness of HuC/D staining. In recent years, there have also been reports of new characteristic pathological findings in HE staining of IG, and diagnostic techniques are in the process of being developed.^4)^ In our cases, we were able to confirm a palisading-like arrangement as reported by Yoshimaru et al.^4)^ In 1997, Ure et al.^5)^ reported 4 (2.8%) cases of IG among 141 cases of ADHD; in 2012, Puri and Gosemann^6)^ reported 10 (5.6%) cases among 178 cases; and in 2015, Ieiri et al.^1)^ reported 15 (4.2%) cases among 355 cases. Although diagnostic techniques, including staining methods, have improved, there seems to be generally no change in the incidence of the disease. If the techniques for identifying IG are further developed, more accurate and faster diagnoses will have a positive impact on future treatment plans.

A 2015 systematic review reported a familial recurrence rate of 7.6% in 4331 HSCR cases.^7)^ Familial onset in HSCR also seems to be rare, but it has been reported in IG as well. Ieiri et al.^1)^ reported that there were 4 patients with IG who had a family history, 2 in other twin babies and 2 in cousins. In the familial occurrence of HSCR, some literature indicates that genetic factors, such as the *RET* gene, may be relevant.^8)^ Henderson et al.^9)^ also conducted a systematic review of twins with HSCR, and they noted that future genetic studies should be performed carefully. Genetic abnormalities in IG have not yet been identified. In addition, there have been reports of familial onset in twins with ADHD, such as intestinal neuronal dysplasia,^10)^ and further studies are needed. Further evidence of familial cases in future studies could help clarify the risks for families of children affected by IG.

## CONCLUSIONS

In our cases, we experienced a very rare instance of familial onset. IG, like HSCR, may exhibit intrafamilial onset. Therefore, early diagnosis and treatment planning are important. Also, providing a careful explanation to the family is essential. We hope that further establishment of the diagnostic methods will enable us to accumulate more cases and provide better treatment in the future.

## DECLARATIONS

### Availability of data and materials

The datasets supporting the conclusions of this article are included within the article.
